# Supplementary material for: Comparative multiomics analysis of cell physiological state after culture in a basket bioreactor
Source: Sci Rep. 2022 Nov 23;12:20161. doi: 10.1038/s41598-022-24687-4 (PMC9686226; doi:10.1038/s41598-022-24687-4)
Supplement: Supplementary file 1 — Supplementary Information 1. [file 41598_2022_24687_MOESM1_ESM.zip › raw data/Transcriptomic raw data/7.AS/2.dasplot/TE1vsTC1/A3SS/17_ZDHHC16_NW_023666048.1_51522364_51522449_+@NW_023666048.1_51523251_51523395_+@NW_023666048.1_51523326_51523395_+.pdf]

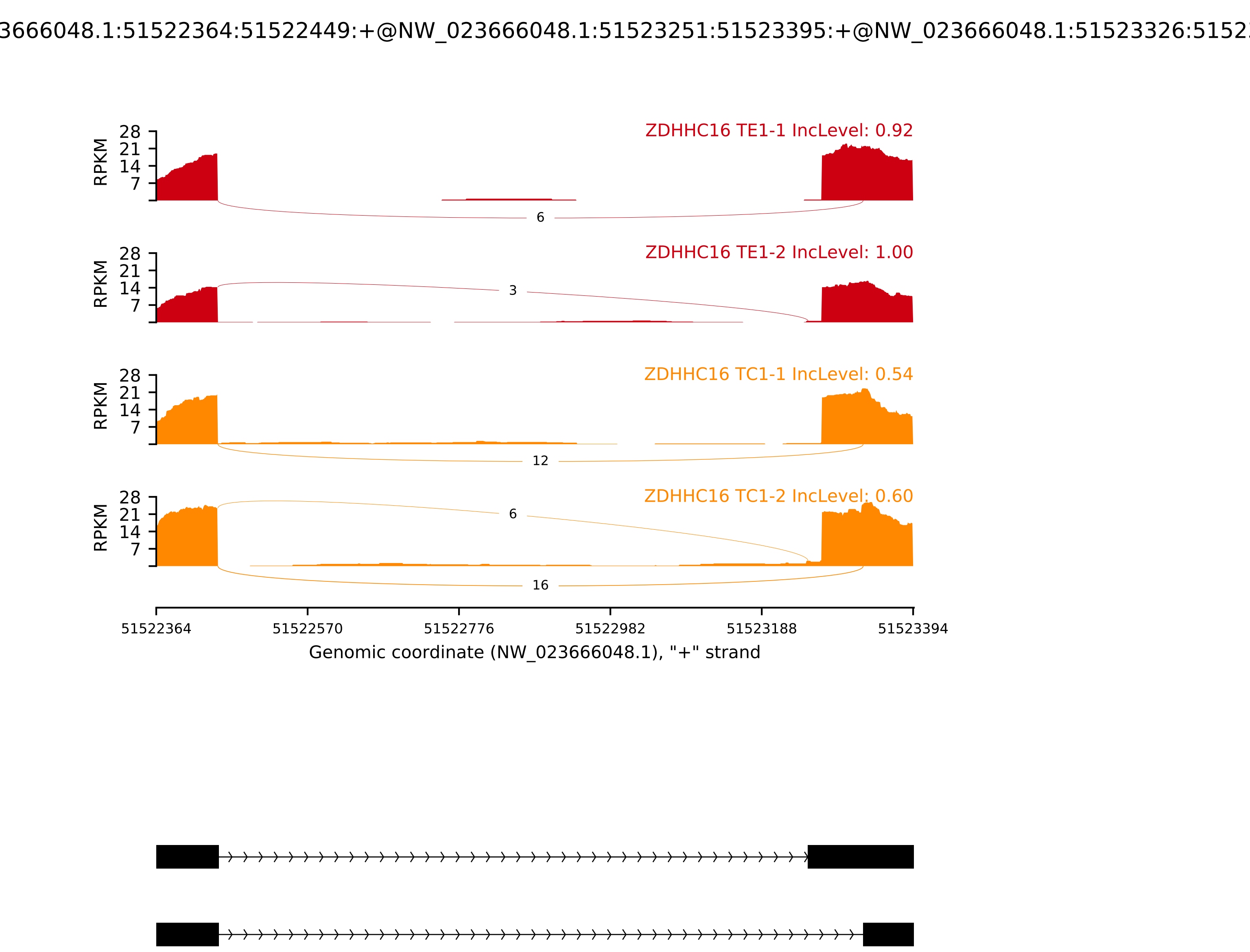
3666048.1:51522364:51522449:+@NW_023666048.1:51523251:51523395:+@NW_023666048.1:51523326:5152
ZDHHC16 TE1-1 IncLevel: 0.92
ZDHHC16 TE1-2 IncLevel: 1.00
ZDHHC16 TC1-1 IncLevel: 0.54
ZDHHC16 TC1-2 IncLevel: 0.60
RPKM
28
21
14
7
6
3
12
6
16
51522364
51522570
51522776
51522982
51523188
51523394
Genomic coordinate (NW_023666048.1), "+" strand
